# Our Contemporaries

**Published:** 1915-01

**Authors:** 


### OUR CONTEMPORARIES.

---

''**The Critic and Guide**'' has absorbed the ''Dietetic and Hygienic Gazette'' which is just completing its thirtieth year. The combined journal will be edited and published by Dr. William J. Robinson, 12 Mt. Morris Park West, New York City. The Jan. issue will be 100,000 copies. We congratulate Dr. Robinson and feel that the readers of the two journals will be greatly benefited by the combination.

---

''**The N. Y. Medical Journal**'' of Dec. 12, calls attention to the scarcity of young doctors in the country. We think the comment should have expressed somewhat more clearly, the fact that the reduced graduation is still far in excess of the needs of the entire population or of any city, but there is in many cases, a lack of available physicians in rural communities and small villages. We have already expressed our willingness to aid in readjusting economic conditions by giving notice of openings for physicians and in finding places—confidentially if desired—for any city physician desiring to enter country practice. Modern inventions and extension of facilities have greatly improved the conditions of country life within the last few years and country practice is prompt in its opportunities for employment and adheres to the old standards of family loyalty.

